# Quantification of cell‐type‐specific plasmodesmata distribution in Arabidopsis roots reveals spatial and patterning dynamics

**DOI:** 10.1111/tpj.70726

**Published:** 2026-02-20

**Authors:** Gwendolyn V. Davis, Jan J. Pavlou, Patrick Li, Marija Smokvarska, Richard S. Smith, Emmanuelle Bayer, George W. Bassel

**Affiliations:** ^1^ School of Life Sciences University of Warwick Coventry CV4 7AL UK; ^2^ CNRS, Laboratoire de Biogenèse Membranaire, UMR 5200 Univ. Bordeaux F‐33140 Villenave d'Ornon France; ^3^ Department of Computational and Systems Biology John Innes Centre Norwich NR4 7UH UK

**Keywords:** plasmodesmata distribution, confocal microscopy, symplasmic connectivity, root patterning, brassinosteroid hormone signalling, plasmodesmata dynamics, root development, quantitative 3D imaging, plasmodesmata pit field spatial patterning

## Abstract

Cell‐to‐cell communication underpins pattern formation and organ function in multicellular organisms. Plant cells can communicate directly through cytoplasmic channels called plasmodesmata. The distribution, abundance, and density of plasmodesmata on plant cell interfaces impact the flow of molecules between plant cells; yet the extent to which these properties are genetically and dynamically regulated remains poorly understood at an organ scale. We developed a quantitative approach to map plasmodesmata pit fields across roots in 3D at cell type and cell interface‐specific resolution. Multiple parameters are captured simultaneously, including plasmodesmata pit field abundance, density, and spatial distribution, enabling parallel multiscale analyses at cellular resolution across this organ. During root maturation, plasmodesmata abundance increases, with the greatest biogenesis occurring within the inner cell layers. This is coupled with changes in the degree of clustering of the pit fields on these inner cell layers: becoming more dispersed on specific cell interface types and more clustered on others. Significant differences in plasmodesmata pit field spatial patterning were detected at cell type‐specific resolution in the *BRASSINOSTEROID INSENSITIVE1* mutant, demonstrating a role for this hormone pathway in channel patterning. The ability to quantify pit field abundance and patterning at cell type‐specific resolution provides novel insight into the developmental and hormonal regulation of potential symplastic connectivity across plant organs, while providing a powerful tool toward the investigation of quantitative systems‐level plasmodesmata distribution and macro‐communication between cells in a complex multicellular system.

## Significance Statement

Plasmodesmata are cytoplasmic channels essential for intercellular communication in plants; yet their organ‐scale distribution and regulation remain poorly understood. Here, we establish a quantitative 3D imaging and analysis framework to map plasmodesmata pit fields at cell‐type resolution in Arabidopsis roots. We show developmental reallocation of pit fields and identify brassinosteroid signaling as a regulator of their spatial patterning. This systems‐level approach provides new insight into dynamic symplastic connectivity and its developmental and hormonal regulation.

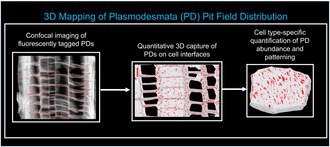

## INTRODUCTION

Cell to cell communication is crucial to multicellularity and plays a key role in cellular patterning and sustaining organ function. Plant cells communicate through a variety of pathways including the extracellular apoplastic pathway, via membrane‐spanning transporters, and directly through cytoplasmic channels called plasmodesmata which form the symplastic pathway (Sager & Lee, 2014).

Plasmodesmata (PD) play a unique role by mediating the direct movement of molecules such as hormones, proteins, and RNAs between cells (Faulkner, 2018). In current models, the opening and closing of plasmodesmata through the deposition and degradation of callose provides a means to dynamically gate cell communication and molecular movement (Faulkner, 2018; Levy et al., 2007). The size exclusion limit (which defines what molecules are able to pass through plasmodesmata) can be modified by deposition of callose at the neck region of the plasmodesmata, allowing symplastic flux to be modulated (Iglesias & Meins, 2000; Levy et al., 2007; Radford et al., 1998; Radford & White, 2001). Therefore, cell‐to‐cell communication through plasmodesmata can be dynamically altered.

Plasmodesmata are often found in clusters known as pit fields, which are located in thinner patches of the cell wall (Carr, 1976; Faulkner, 2018). It has been proposed that the distribution of plasmodesmata pit fields also impacts the capacity of molecules to pass through these channels (Deinum et al., 2019). Modeling approaches indicated that the clustering of plasmodesmata into pit fields can limit molecular movement (Deinum et al., 2019).

The communication between plant cells through plasmodesmata is therefore impacted by each of the abundance, distribution, and patterning of these channels on cell interfaces. The ability to simultaneously measure each of these properties at an organ scale would therefore provide insight into how global cell communication can take place within plant organs.

A variety of methods capable of mapping plasmodesmata have been developed (Sankoh & Burch‐Smith, 2021). These approaches vary in terms of both the scale and the resolution with which they can image plasmodesmata, representing a trade‐off in the information that can be obtained.

Transmission Electron Microscopy (TEM) provides very high‐resolution detail of plasmodesmata structure and is also useful toward identifying the distribution in 2D sections (Waigmann et al., 1997). The abundance of plasmodesmata can, however, be underestimated using this thin layer imaging approach, owing to the limited sampling of tissues into sections.

Toward more accurate mapping of plasmodesmata abundance and distribution, 3D approaches have been developed. These include the use of focused ion beam‐scanning electron microscopy (FIB‐SEM) (Deng et al., 2023), serial block face‐scanning electron microscopy (SBF‐SEM) (Paterlini et al., 2020; Reagan et al., 2017), and TEM tomography (Nicolas et al., 2017; Yan et al., 2019). These methods provide high‐resolution insights into plasmodesmata distribution within a portion of a plant tissue. They are however limited in throughput due to the time required for fixation, embedding, and sectioning, and they are limited to small sample sizes. An additional drawback of these sectioning‐based approaches, except for FIB‐SEM and SBF‐SEM, is the loss of information between slices, which limits the ability to accurately map plasmodesmata in 3D. The large‐scale distribution of plasmodesmata remains difficult to examine using such techniques.

Previous studies have shown that plasmodesmata are rarely, if ever, symmetrically distributed around a cell (Juniper & Barlow, 1969). It is important for the pit fields of all interfaces of a cell to be examined. The same study (Juniper & Barlow, 1969) examined the distribution of plasmodesmata in the root cap and the first 0.5 mm of the primary root of *Zea mays* variant White Horse Tooth using electron microscopy. They determined that apical–basal interfaces (referred to there as transverse walls) possessed a larger average number of plasmodesmata than circumferential and radial interfaces (referred to there as longitudinal walls). They also found that as cells move from the zone of division, the ratio of the number of plasmodesmata on the apical–basal interfaces to those on radial interfaces increases (Juniper & Barlow, 1969).

Confocal microscopy presents a rapid approach to map pit field distribution. While this comes at the expense of resolving individual plasmodesmata, it benefits from having a higher throughput and larger scale coverage of samples than the high‐resolution single plasmodesmata‐resolving methods (Fitzgibbon et al., 2013). These approaches use proteins localized to plasmodesmata pit fields tagged with fluorescent proteins. An image analysis pipeline to quantify plasmodesmata distribution and abundance has been developed for the leaf epidermis (Fitzgibbon et al., 2013), which enabled a larger portion of a sample to be imaged. This, however, comes at the cost of mapping plasmodesmata in 2D and using only the most accessible outer portion of living leaf samples.

We developed a confocal‐based approach to map plasmodesmata pit field distribution in 3D at cell interface‐specific resolution across plant organs. Using clarified tissue with fluorescent reporters, this method enables high‐resolution imaging deep into roots with relatively low cost and standard equipment. This is coupled with computational analysis (de Reuille et al., 2015; Strauss et al., 2022) that quantifies pit field abundance, density, and spatial patterning across development and signaling contexts. This scalable framework complements ultrastructural techniques, extending plasmodesmata research to organ‐level dynamics and function.

## RESULTS

### Imaging and analysis of cell interface‐specific plasmodesmata pit field abundance in Arabidopsis roots

**Figure 1:**
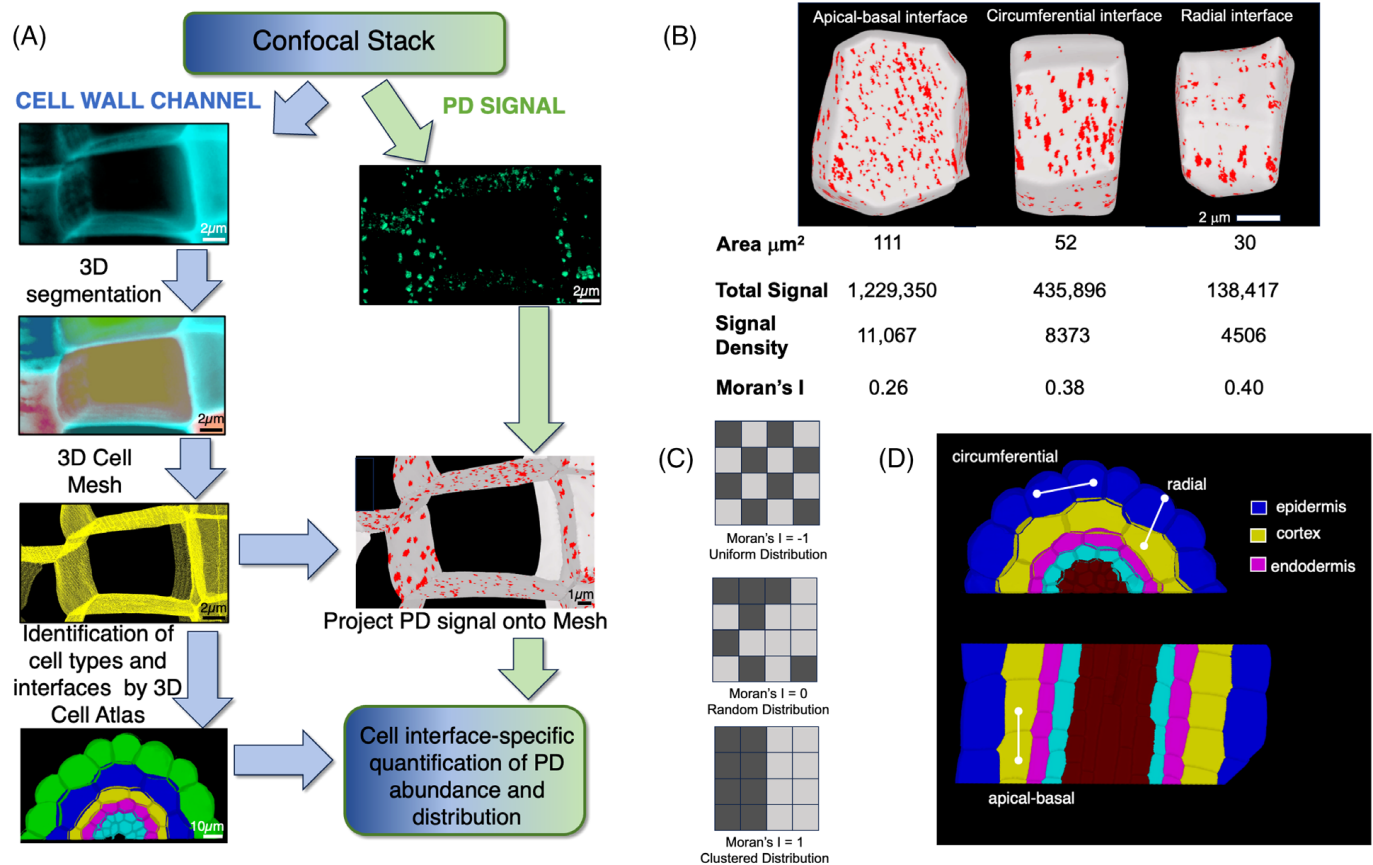
The workflow for quantification of PD pit field distribution in 3D using confocal microscopy. (A) Image acquisition and analysis pipeline used to perform organ scale cell type‐specific analyses of plasmodesmata abundance across root cell interfaces. (B) Visual representation of a root epidermal cell with plasmodesmata signal projected onto the cell surface. The various types of data acquired for each cell interface are listed below. Total Signal represents the total plasmodesmata marker signal on the interface. Signal Density is the Total Signal divided by the interface surface area. For comparison between samples imaged on different days with different expression, these values were expressed as percentages of the Total Signal of all the interfaces in the sample, allowing distributions to be compared. (C) Moran's I is a statistical measure of spatial distribution. Schematic illustration of the patterns and values generated using Moran's I. (D) Illustrations of the labeling of cell interface types within the root: circumferential, apical–basal, and radial interfaces.

To quantify plasmodesmata pit field abundance at cell–cell interface resolution, an integrated imaging and computational approach was developed based on confocal microscopy (Figure 1A). Proteins tagged with fluorescent reporters were targeted to plasmodesmata; pit fields were imaged, and fluorescence above a critical threshold was used as a proxy to define the abundance of plasmodesmata pit fields on cell interfaces.

A cell wall stain in a different channel to the plasmodesmata signal was used to capture cell shape and perform 3D cell segmentation. The plasmodesmata signal was then projected onto the mesh representing cell shapes and captured on cell interfaces where adjacent cell meshes were in physical contact. A series of distinct analyses quantifying pit field abundance and distribution were able to be performed simultaneously within MorphoGraphX using these multidimensional 3D cellular resolution organ scale datasets (Figure 1B).

*Total Signal*: The total plasmodesmata pit field signal on an interface. This represents the abundance of plasmodesmata pit fields on a given cell interface. This acts as a proxy for the total capacity for symplastic communication between pairs of cells. When comparing between samples, Total Signal is represented as a percentage of the Total Signal of all the interfaces in the entire sample. This allows comparison between different samples imaged on different days with various expression levels when lacking an internal control.

*Signal Density*: The Total Signal of plasmodesmata pit fields on an interface divided by the surface area of that interface. This refers to the amount of plasmodesmata pit fields per unit area on cell interfaces, or the concentration of intracellular channels. Expressed as a percentage, Signal Density describes this value as a percentage of the total signal for the entire sample allowing comparison between different samples imaged on different days.

*Moran's I*: Finally, a measure of spatial autocorrelation called Moran's I was able to examine how the pit field density is distributed across the cell interfaces (Moran, 1950) (Figure 1C). Moran's I examines the uniformity with which an entity is distributed across a surface. A perfectly non‐uniform distribution such as a checkers board gets a value of −1 while a half and half clustered segregated distribution gets a value of +1, and intermediates their values in between. This can be used to understand the degree to which plasmodesmata pit fields are clustered on cell interfaces.

These analyses were conducted for every interface within a section of root tissue imaged, and cell type and interface type identified. Cell types were categorized by layer of the root, and epidermis, cortex, and endodermis cells were analyzed. Interface types were identified as apical‐basal, circumferential, and radial (Figure 1D).

**Figure 2:**
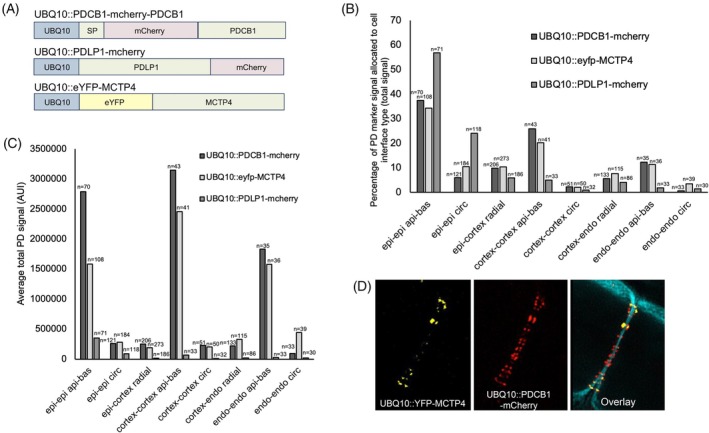
Comparison of PD markers for pit field quantification. (A) Schematics of PD marker constructs used in this study. MCTP4, MULTIPLE C2 DOMAIN AND TRANSMEMBRANE REGION PROTEIN 4; PDCB1, PLASMODESMATA CALLOSE‐BINDING PROTEIN 1; PDLP1, PLASMODESMATA LOCALIZED PROTEIN 1; SP, signal peptide; UBQ10, UBIQUITIN10 promoter. (B) Percentage of Total Signal for respective reporter constructs allocated to each cell interface interaction type (C) Same as (B) using absolute signal collected from the microscope in Arbitrary Intensity Units, error bars show standard deviation. (D) Super resolution SIM imaging of the PDCB1 and MCTP4 markers on an epidermal cell interface in the same cells. Arabidopsis root meristem zone was imaged and examined. The cell wall is shown in blue. *n* = the number of interfaces of the cell interface type examined.

Three different core plasmodesmata proteins known to be targeted to these channels (Brault et al., 2019; Johnston et al., 2023; Miras et al., 2022) were fused to fluorescent proteins and examined to identify a marker suitable for labelling these structures using confocal imaging and the quantification pipeline. To select the most suitable marker for mapping plasmodesmata pit fields, proteins were placed under the UBIQUITIN10 (UBQ10) promoter and stably transformed into Arabidopsis. Constructs examined included PLASMODESMATA CALLOSE‐BINDING PROTEIN 1 (PDCB1) (Simpson et al., 2009) with an internal mCherry tag after the signal peptide, PLASMODESMATA LOCALIZED PROTEIN 1 (PDLP1) (Thomas et al., 2008) with a C‐terminal mCherry tag, and MULTIPLE C2 DOMAIN AND TRANSMEMBRANE REGION PROTEIN 4 (MCTP4) (Brault et al., 2019) with an N‐terminal YFP tag (Figure 2A).

While a broadly similar total signal distribution was observed for all three constructs (Figure 2B,C), the PDLP1 showed low signal intensity and was not suitable for the long exposure times required for high‐resolution image acquisition (Figure 2C). Super resolution examination of the PDCB1 and MCTP4 constructs in the same plant using Structured Illumination Microscopy revealed PDCB1 to label more wall‐associated structures than MCTP4 (Figure 2D). We did not detect phenotypes in the UBQ10::PDCB1‐mCherry line with respect to root growth or flowering time (Figure S3A,B). PDCB1 was therefore identified as the most reliable reporter for high‐resolution, organ‐scale quantification of plasmodesmata distribution.

### Cell type‐specific plasmodesmata pit field abundance and density in the Arabidopsis root

Root tissue was selected based on the ability to comprehensively image the cells of this organ, our ability to computationally identify cell types in the tissue (Montenegro‐Johnson et al., 2015), and our detailed understanding of the development of this organ (Petricka et al., 2012; Zhu, Lucas, & Rost, 1998).

**Figure 3:**
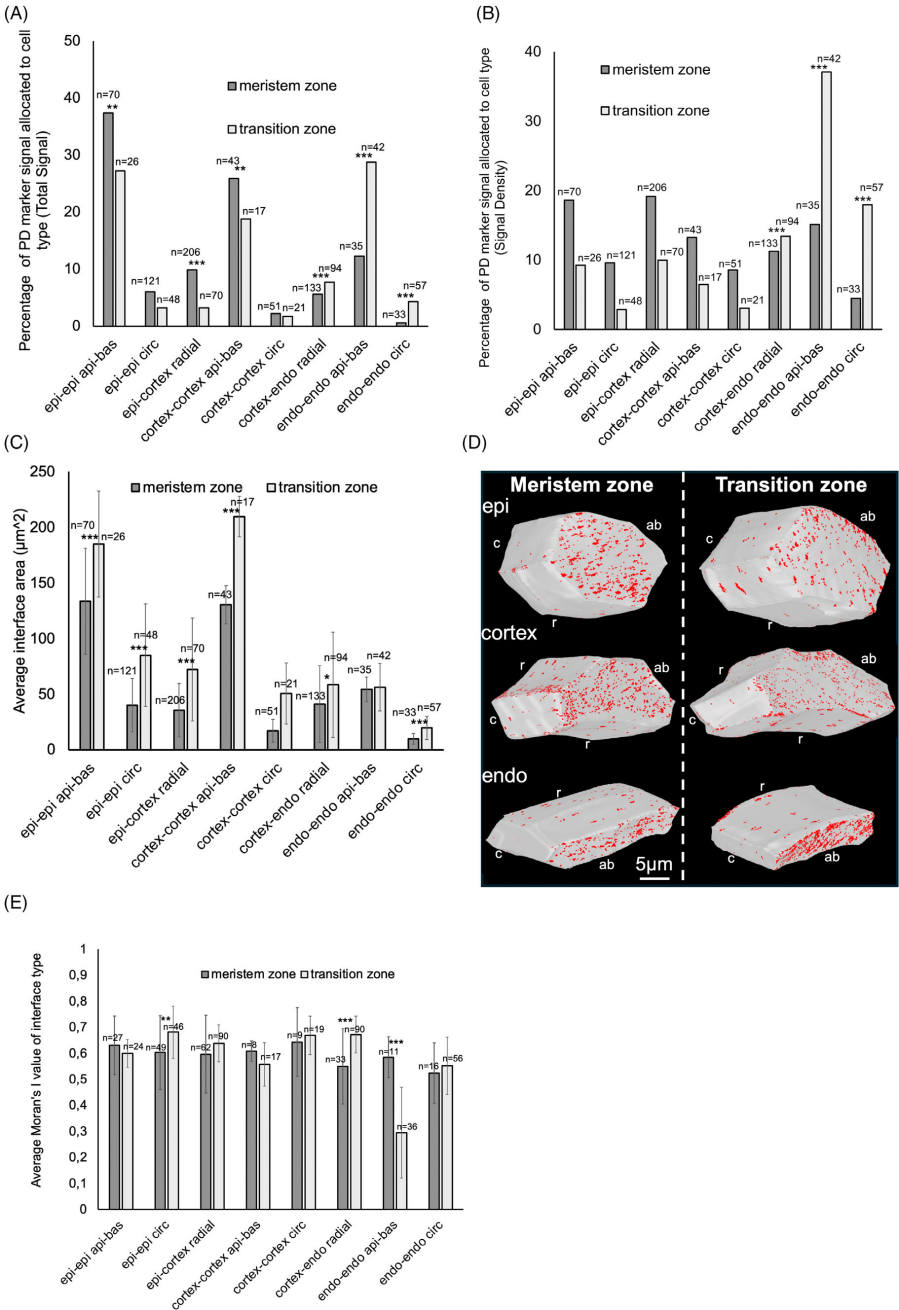
Quantitative analysis of PD pit field distribution in root meristem and transition zone. (A) Changes in the Total Signal of UBQ10::SP‐mCherry‐PDCB1 between the root meristem and transition zone of the same individual (B) Same as (A) for Signal Density. (C) Average cell interface size for each cell interface in the meristem and transition zones of the root. (D) 3D rendered cells with UBQ10::SP‐mCherry‐PDCB1 signal projected onto their surfaces for each cell type investigated. Cell interface orientation was annotated using letters: abaxial–adaxial (ab), circumferential (c), and radial (r). (E) The average Moran's I value for signal dispersion distribution for each cell interface interaction type. Error bars show standard deviation. Asterisks following *t*‐tests indicate significance levels of *0.01, **0.005, and ***0.001. *n* = number of interfaces in sample of each cell interface type.

**Figure 4:**
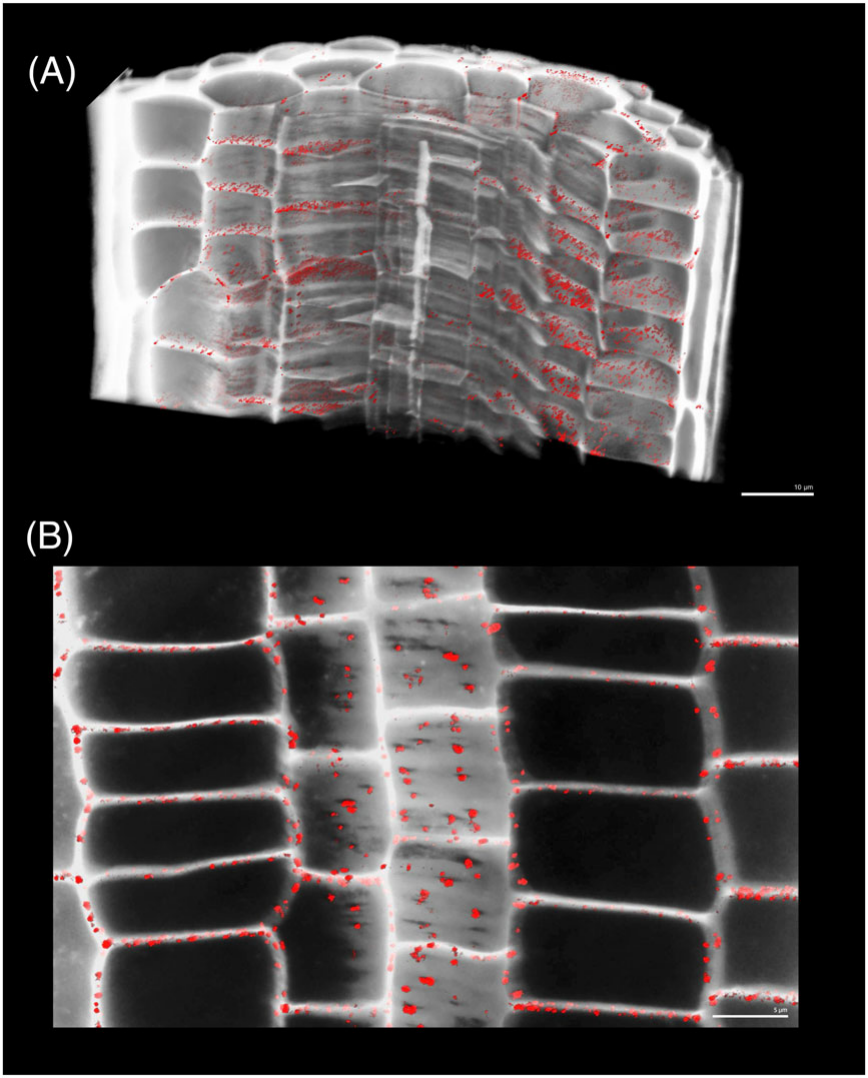
Confocal stacks from the Arabidopsis root showing PD pit fields on cell interfaces. (A) a section of a stack of Arabidopsis root tissue, with the cell wall in gray and the plasmodesmata pit field signal in red. (B) A thin optical section of a stack of Arabidopsis root tissue. Thinning in the cell wall has been shown to be associated with the location of plasmodesmata pit fields. Here, such thinning appears as holes in the cell wall signal, which the plasmodesmata pit field signal slots into. Pit field signal can be seen overlaying cell interfaces.

The cell type‐specific distribution of UBQ10::SP‐mCherry‐PDCB1 across the Arabidopsis root meristem was examined (Figure 3A–E; Figure S1A,B). We found the greatest Total Signal on the apical–basal interfaces across all cell layers of the root (Figure 3), which can also be observed visually (Figure 4).

The total abundance of plasmodesmata pit fields on radial cell interfaces was relatively lower as in the circumferential cell direction. These distribution patterns and relative amounts of plasmodesmata pit fields were consistent with previous literature measuring numbers of plasmodesmata using TEM (Zhu, Lucas, & Rost, 1998; Zhu, O'quinn, et al., 1998).

When considering the interface sizes, the distribution of Signal Density is more broadly distributed across the different cell types and cell interfaces (Figure 3B). These data suggest the greatest total capacity for symplastic flow is along cell files of the outer cell layers, while there are biases toward greater plasmodesmata pit field abundance in the inner cell layers where the cell interface sizes are relatively smaller.

An advantage of full 3D imaging is the capture of circumferential interfaces which are difficult to capture using 2D approaches. This identified the presence of a capacity for symplastic communication within cell types of the Arabidopsis root. It was shown by TEM examination of the primary root meristem of Arabidopsis that apical basal interfaces have a much greater plasmodesmata frequency than radial or circumferential (Zhu, Lucas, & Rost, 1998; Zhu, O'quinn, et al., 1998). Our results are in agreement with these findings (Juniper & Barlow, 1969; Zhu, Lucas, & Rost, 1998; Zhu, O'quinn, et al., 1998), with apical–basal interfaces having the greatest plasmodesmata abundance (Total Signal) for all cell types. However, there is measurable pit field signal on both the radial and circumferential interfaces. It is possible that previous studies have underestimated the symplastic connectivity on such interfaces, that our 3D approach is able to more thoroughly capture.

### Cell type‐specific plasmodesmata pit field abundance and density changes across root development

**Figure 5:**
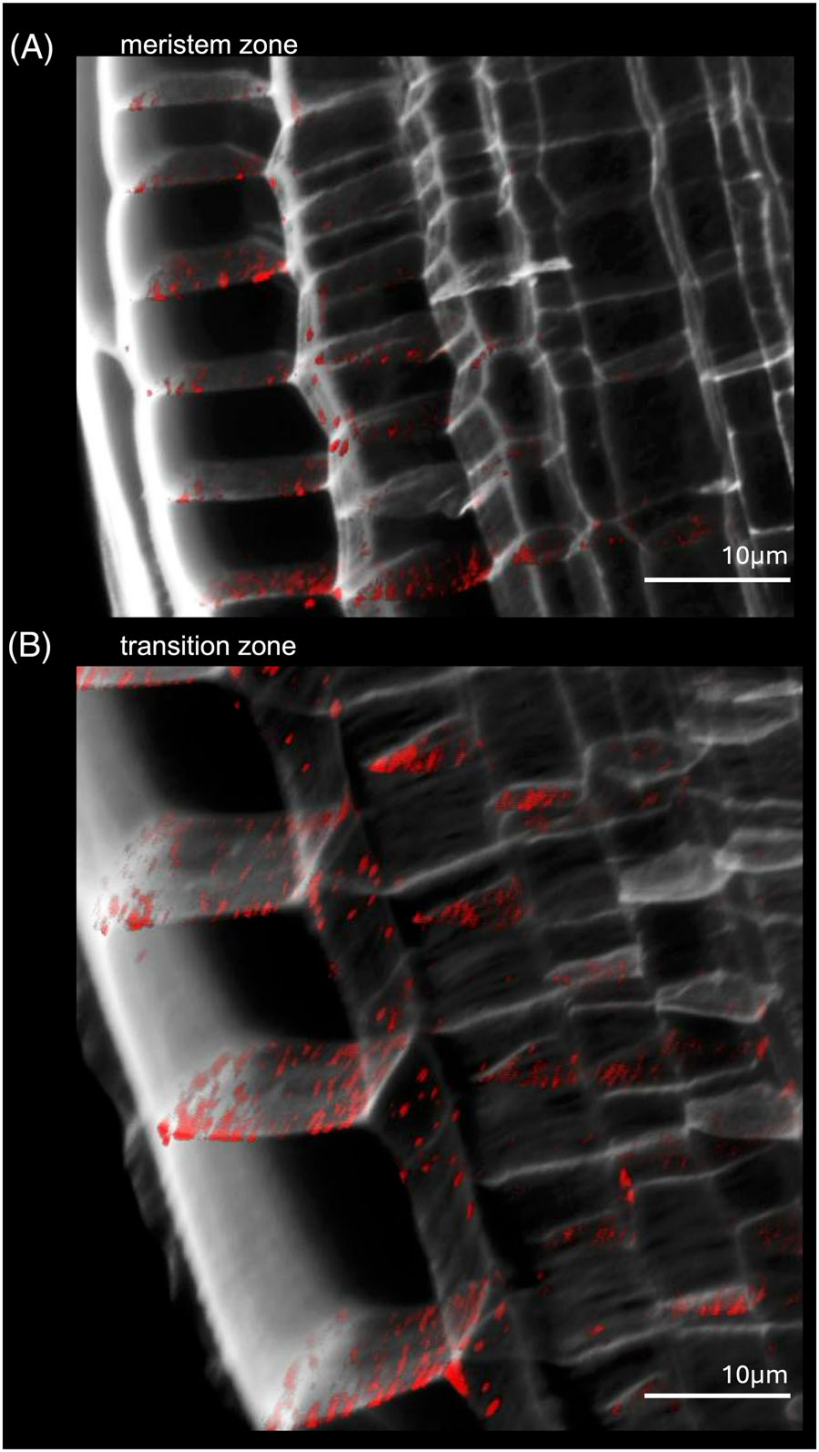
Sections of confocal stacks of meristem zone and transition zone of an Arabidopsis root. (A) A section of a stack of Arabidopsis root tissue in the meristem zone. The cell wall is in gray, and the plasmodesmata pit field signal is red labeled by a fluorescent marker tagged to the PDCB1. (B) The same, but in the transition zone. The stacks were taken of the same root consecutively in the same imaging session.

Relative changes in plasmodesmata pit field Total Signal and Signal Density across root development were examined at cell type‐specific resolution by imaging the meristem and transition zone in the same root. Stacks imaging the plasmodesmata distribution within the meristem zone and transition‐elongation zone of the same Arabidopsis root were collected (Figure 5). These were then investigated quantitatively. Total Signal showed significant proportional increases toward the endodermal cell interfaces across root development, while concurrent significant proportional decreases on outer cell interfaces were identified (Figure 3A). These changes were not matched by absolute changes in signal abundance on cell interfaces (Figure S1A).

The Signal Density of pit fields also increased on the endodermal cell interfaces across development, while significant changes in the outer cell layers were not observed (Figure 3B). This likely reflects the greater and significant increases in cell interface size on the outer cell layers than the inner cell layer interfaces (Figure 3C) and is supported by there being limited significant changes in absolute pit field density signal (Figure S1B).

During root development, there is therefore a progressive shift in increased pit field Total Signal and Signal Density toward the cell interfaces in the inner cells of the root relative to the outer cell layers. Concurrently, relative decreases in pit field Signal Density are driven by cell expansion and increases in cell interface area (Figure 3B,C).

The general pattern of pit field distribution found in this data are consistent with previous studies, with a greater quantity of pit fields found on apical–basal interfaces along the root relative to circumferential and radial interfaces (Figure 3; Figure S1A,B) (Juniper & Barlow, 1969; Zhu, Lucas, & Rost,^1998^).

From examination of the Moran's I values, we find there is greater clustering of PD into pit fields at the cortex–endodermis radial interfaces in the transition zone relative to the meristem (Figure 3E). Previous literature has described a general increase in clustering of plasmodesmata into pit fields alongside cell elongation in multiple species, to differing degrees (*Trifolium repens* L., *Raphanus sativus* L., *Z. mays* L., *Sorghum vulgare* L.) (Seagull, 1983). Our results therefore align with this prior data, indicating the same process likely occurs in Arabidopsis root elongation.

Our data show this increased clustering in pit fields coincides with an increase in signal density and total signal on the cortex–endodermis radial interfaces. Previously, work reported that plasmodesmatal frequency (roughly equivalent to our measure of Signal Density) does not decline as a result of cell elongation, and in fact increases in frequency and clustering concurrently as a result of secondary PD formation in preexisting pit fields of primary plasmodesmata (Seagull, 1983). These results are consistent with previous reports, and likely this process is similar in multiple plant species.

On the endodermis apical–basal interfaces, however, the plasmodesmata pit fields are significantly more dispersed in the transition zone compared to the meristem zone. Despite a proportionally greater Total Signal and Signal Density, the clustering in pit fields is reduced. Perhaps *de novo* secondary PD formation is more prominent on such interfaces, rather than secondary PD formation by budding from previously established pit fields.

While other interface interaction types in the epidermis and cortex showed differences in Total Signal (Figure 3A), only the epidermal circumferential interfaces were slightly significantly different in Moran's I value (Figure 3E) between meristem zone and transition zone. The proportion of Total Signal representing plasmodesmata pit fields was altered on some interfaces between meristem and transition zone, but the patterning of plasmodesmata pit fields remained the same across these two zones of the root on the outer cell layers.

### The brassinosteroid hormone signaling pathway has the capacity to regulate plasmodesmata pit field abundance, density, and distribution on defined cell interfaces in the root meristem zone

Hormone signaling pathways have been shown to impact the opening and closing of plasmodesmata through the modulation of callose levels and gene expression associated with its synthesis and degradation. Examples of this include ABA (Mehra et al., 2022) and brassinosteroids (Wang et al., 2023). By contrast, little is known about how these pathways impact plasmodesmata abundance and distribution, also whether hormone signaling acts to reshape the symplastic pathway beyond the control of plasmodesmata aperture.

**Figure 6:**
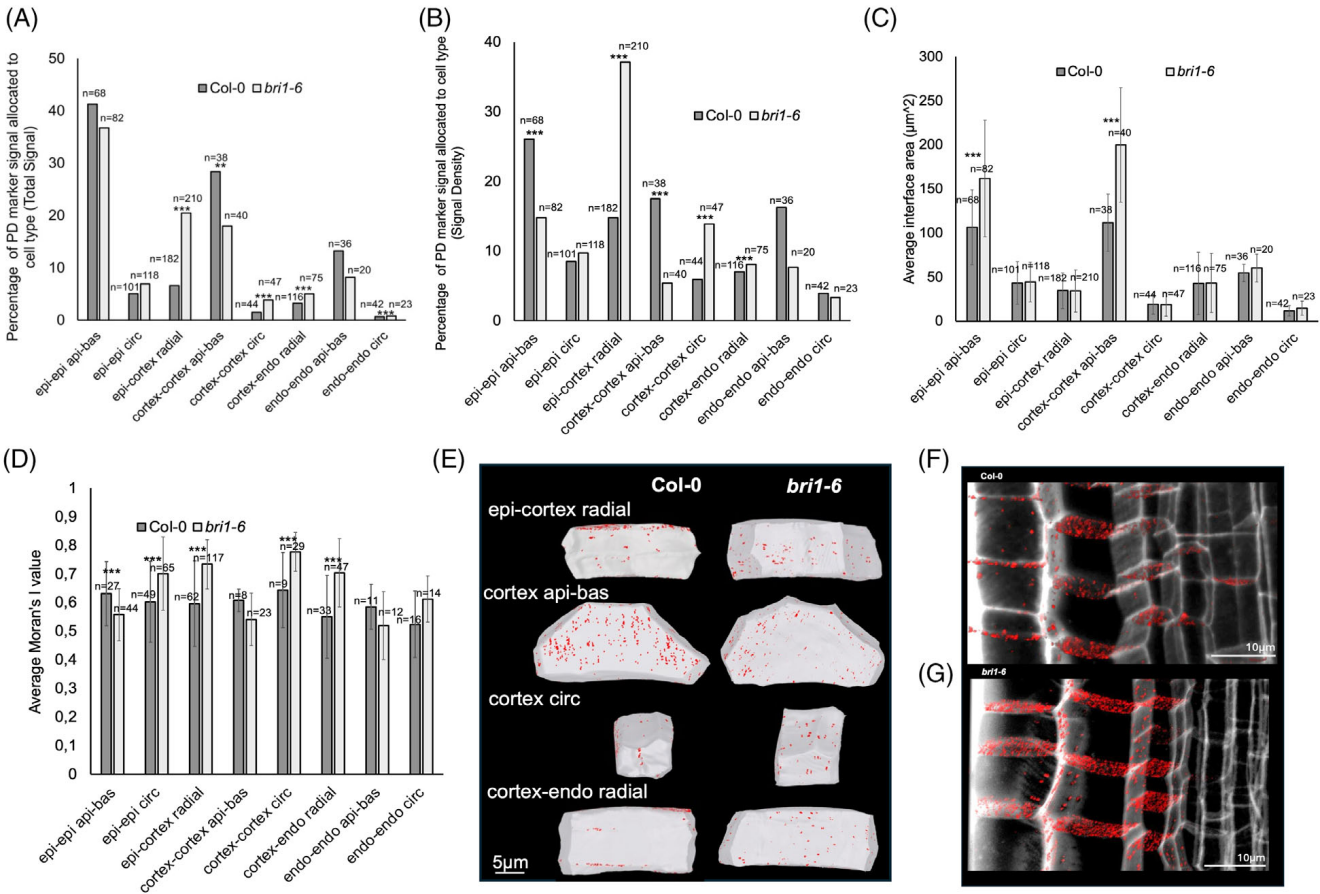
Quantitative analysis of PD pit field distribution in Col‐0 and *bri1‐6* root meristem zone. (A) Percentage Total Signal and (B) percentage Signal Density in an individual of the brassinosteroid *bri1‐6* mutant root meristem region. (C) Average interface area of Col‐0 sample compared to *bri1‐6* mutant root meristem region. (D) Moran's I analysis of the spatial distribution of plasmodesmata on the cell interfaces of the *bri1‐6* mutant. (E) Selected 3D rendered cells from respective mutants which showed significant differences in UBQ10::SP‐mCherry‐PDCB1 signal abundance or density with plasmodesmata signal projected onto their surface. (F) A section of a stack of Arabidopsis root tissue, with the cell wall in gray and the plasmodesmata pit field signal in red labeled by fluorescent marker tagged to the PDCB1, expressed in Col‐0. (G) The same, but expressed in the *bri1‐6* mutant. Error bars show standard deviation where applicable to plots. Asterisks following *t*‐tests indicate significance levels of *0.01, **0.005, and ***0.001. *n* = number of interfaces in sample of each cell interface type.

Brassinosteroid signaling is promoted by the receptor *BRASSINOSTEROID INSENSITIVE 1* (*BRI1*). The recessive *bri1‐6* mutant has diminished response to this hormone and showed significant relative increases in Total signal and Signal Density on radial cell walls, and a significant relative decrease in Signal Density on the apical–basal interface of the epidermis (Figure 6A,B; Figure S2A,B). Brassinosteroids are therefore capable of modulating plasmodesmata abundance and density in addition to plasmodesmata aperture (Wang et al., 2023).

The *bri1‐6* mutant interface area was also examined in comparison with Col‐0. The average interface area was significantly different for the apical–basal interfaces of the epidermis and the cortex; however, other interfaces were not significantly different in terms of average interface area (Figure 6C). This coincides with significant differences in PD pit field Signal Density on these interfaces. This may explain the discrepancy on the epidermal apical–basal interfaces between Signal Density and Total Signal: proportionally the same Total Signal was recorded; however, it was spread across a large average interface area, resulting in a significantly reduced relative Signal Density in the *bri1‐6* mutant. This makes even more notable the observation of increased Total Signal and Signal Density on the Cortex apical–basal interfaces, as these interfaces have a significantly larger average area with also a greater relative pit field concentration.

The patterning of plasmodesmata pit fields in the *bri1‐6* mutant was examined using Moran's I (Figure 6D). The mutant was found to have a significantly higher value on multiple interfaces and a lower value on the apical–basal interface on the epidermis. With most interfaces showing an increase in Moran's I, this suggests the brassinosteroid signal controls plasmodesmata distribution within pit fields in the root by dispersing these channels and reducing their clustering within pit fields. These patterns have been proposed to impact symplastic transport, whereby plasmodesmata clustered in pit fields reduce the predicted permeability of plasmodesmata (Deinum et al., 2019).

Differences in plasmodesmata pit field distribution can be challenging to observe merely by eye or in a purely qualitative manner. Subtle differences in pit field shape and distribution can be visually observed (Figure 6E–G), however, this technique allows differences in distribution to be quantified and understood to a much greater degree.

## DISCUSSION

Plasmodesmata (PD) have long been recognized as central mediators of symplastic communication, yet most studies have focused either on ultrastructural features of individual channels or on local observations in restricted tissue regions (Faulkner, 2018; Juniper & Barlow, 1969; Zhu, Lucas, & Rost, 1998; Zhu, O'quinn, et al., 1998). These pioneering studies established that PD are unevenly distributed across cell interfaces and that their abundance differs between apical–basal and radial walls, but the organ‐scale plasticity of PD allocation remained unresolved. Efforts to map PD distributions using TEM, FIB‐SEM, and SBF‐SEM revealed structural detail (Deng et al., 2023; Nicolas et al., 2017; Waigmann et al., 1997); yet throughput and sample size limitations constrained quantitative analyses across tissues and developmental stages. Leaf‐level confocal approaches partially addressed this by introducing pit‐field–localized fluorescent reporters (Fitzgibbon et al., 2013), but these remained limited to surface‐accessible tissues and two‐dimensional sampling.

The present study builds directly on these foundations by providing the first three‐dimensional, cell type–resolved quantification of PD pit field abundance and spatial patterning across an entire organ. By integrating fluorescently tagged PD markers with image analysis pipelines (de Reuille et al., 2015; Strauss et al., 2022), we capture multi‐scale parameters—total abundance, density, and spatial clustering—that could not previously be measured simultaneously. This establishes a framework for quantitatively evaluating how PD allocation contributes to developmental patterning and signaling capacity.

Our data demonstrate that PD distribution is not static but undergoes developmental reallocation, with progressive enrichment of pit fields in inner root layers during maturation. Prior ultrastructural studies suggested that secondary PD biogenesis could contribute to altered symplastic connectivity during elongation (Brunkard & Zambryski, 2017; Ehlers & Kollmann, 2001). The observed shifts in both abundance and clustering across developmental zones are consistent with this model, now resolved quantitatively at the organ scale. Similarly, previous work across multiple species reported increased PD clustering during cell elongation (Overall & Blackman, 1996), and we extend this to Arabidopsis roots, showing that clustering can be interface‐specific and developmentally regulated. It is also possible that this result could instead reflect pre‐patterning for Casparian strip formation. The Casparian strip forms in the endodermis of Arabidopsis roots, and it has been shown that close to the meristem (within transition zone and early elongation zone), Casparian strips already form a functional endodermal barrier (Alassimone et al., 2010). It is possible that the observed shifts in PD pit field distribution could reflect pre‐patterning for the formation of the Casparian strip further along in the differentiation zone of the root.

In addition to developmental dynamics, we indicate the possibility of hormone‐dependent regulation of PD patterning, although confirmation of such regulation would require further study. Hormone signaling pathways were previously linked to PD aperture via callose turnover (Levy et al., 2007; Mehra et al., 2022; Wang et al., 2023), but whether hormone signaling influenced PD abundance and distribution was unclear. The finding that brassinosteroid signaling via BRI1 has the capacity to modify both PD density and spatial dispersion reveals a possible additional layer of regulation, suggesting that symplastic connectivity can be reshaped not only by gating but also by changes in channel allocation. This expands the functional scope of hormone action in tissue coordination and highlights the potential of PD as integral components of developmental signaling networks. However, it should be noted that while these results propose exciting possibilities, further study is required to confirm them; the *bri1‐6* mutant is known to show growth defects, and so alterations in PD pit field distribution may also be in part a consequence of secondary effects of this phenotype, even though all except for two cell interface types were not shown to have significant changes in interface area relative to Col‐0. It is also possible that untangling PD pit field distribution and growth changes may be a challenge in itself as such processes may be so intimately intertwined.

This proposed workflow has strengths for the study of PD pit field distribution shifts; however, it is not without constraints. As previously mentioned, confocal microscopy cannot resolve individual PD but only pit fields; also, it is limited in its ability to draw conclusions about PD quantity due to it not being possible to identify PD or pit field number. In order to gather that kind of data, this workflow would need to be employed alongside other, more high‐resolution microscopy techniques. It should also be noted that while this technique is faster than many high resolution approaches (such as FIBS‐SEM), it does still take time for both the gathering of confocal stacks and the subsequent analysis. While individual samples take time, the strength of this technique is in the sheer number of cells, interfaces, and interface area that can be analyzed in a 3D manner. If utilized alongside other approaches, this could be a very powerful technique for giving a large overview of PD distribution and identifying general trends. Integration of different techniques for the imaging of plasmodesmata is a current and ongoing challenge within the field but also the route forward to a fuller understanding of PD distribution, form, and function.

It is possible that with some refinement, this workflow could also be applied to the study of other plant tissues, such as lateral root, fully differentiated root, or the shoot apical meristem (SAM). The SAM has a CellAtlas available within MorphoGraphX (Montenegro‐Johnson et al., 2019), so this approach would be especially possible. Provided a plant tissue can be fixed and cleared successfully, and a fluorescent PD marker expressed, then this workflow is possible and could be used alongside other PD imaging techniques or alone as a study of general trends of PD pit field distribution.

Taken together, this work advances the field by moving beyond static or local descriptions of PD to quantitative, system‐wide analyses. The ability to measure PD distribution across tissues in three dimensions establishes new opportunities for linking symplastic connectivity with gene expression domains, mechanical constraints, and hormone gradients. By demonstrating both developmental and hormonal plasticity of PD allocation, we provide a framework for interrogating how intercellular communication scales from local channel structure to whole‐organ information flow. This approach can be integrated with ultrastructural imaging and functional assays, positioning PD research for systems‐level dissection of communication in complex multicellular contexts.

## METHODS

### Plant growth conditions

Arabidopsis plants of the ecotype Colombia (Col‐0) were grown under glasshouse conditions with 16 h light (23°C) and 8 h dark at 22°C. Hormone signaling mutant plants were also grown under these conditions. Seeds were harvested and stored for 1 month prior to use. Mutant used in this study was *bri1‐6* (NASC N399).

### Development of fluorescent markers targeted to plasmodesmata

Genetic constructs utilized were sourced from Col‐0 genomic DNA. The promoter sequences were incorporated into the pDONR‐p4RP1 vector, while the genes and fluorescent tags were incorporated into either the pDONR221 or pDONR‐P2RP3 vectors, employing the MultiSite‐Gateway cloning system. The three constructs, including UBQ10::PDCB1‐mCherry, UBQ10::eYFP‐MCTP4 (Brault et al., 2019), and UBQ10::PDLP1‐mCherry, were subcloned into destination vectors pH7m34GW or pB7m34GW by the LR recombination system (Karimi et al., 2002). The expression vectors were introduced into Agrobacterium strain GV3101, which was subsequently utilized for the transformation of Arabidopsis via the floral dip method (Clough & Bent, 1998). Selection of transformed seeds was performed based on resistance to Hygromycin.

### Sample preparation

Seeds were sterilized and plated on vertical 1% (w/v) agar media containing ½ MS and grown for 7 days. Root tissue was fixed in 4% (w/v) paraformaldehyde for 40 min. Fixed tissue was then washed twice with Phosphate Buffered Saline and then cleared using the ClearSee protocol (Kurihara et al., 2015, 2021). Tissue was cleared for at least 2 days before imaging.

### Image acquisition

Plant tissue carrying fluorescent proteins targeted to plasmodesmata pit fields (Figure 2) was fixed and cleared following the ClearSee protocol (Kurihara et al., 2015, 2021). Following tissue clearing, cell walls were stained using 0.001% (v/v) SCRI Renaissance Stain 2200 (SR2200, Renaissance Chemicals), and samples were placed in a 35 mm Cellview Cell Culture Dish (Greiner, United Kingdom). Clarified roots were subjected to multi‐channel confocal imaging with the cell wall collected in one channel, and the plasmodesmata pit field fluorescence collected simultaneously in the second channel.

Images were acquired using an inverted Zeiss LSM880 confocal microscope with a high NA 63x objective. Cell wall was excited using the 405 nm laser detected 420–500 nm, eYFP was excited using the 488 nm laser with detection centered around the peak emission of 527 nm, and mCherry was excited using the 561 nm laser with detection centered around the peak emission at 610 nm. Plasmodesmata pit field signal was collected using the GaAsP (Gallium Arsenide Phosphide) detector. Acquisition of stacks took roughly 40 min of imaging time.

### Cell segmentation and identification of cell types and interfaces

Cells were segmented in 3D by first applying a Gaussian blur radius 0.5 followed by an ITK autoseeded watershed to segment the cells of tissues in 3D (www.itk.org) as previously described (Bassel et al., 2014; de Reuille et al., 2015). Segmented cells were manually inspected and corrected to ensure accuracy (Bassel, 2015).

3D polygonal meshes describing cell surfaces were created using the 3D Marching Cubes algorithm, with a cube size 0.5 for all analysis, except for spatial analyses using Moran's I (Moran, 1950) which used a cube size of 0.2. A total of 10 smooth passes were applied to the mesh at the time of the creation to preserve cellular connectivity (Bassel, 2015). Meshes were used to identify cell interactions and interface surface area (Jackson et al., 2017, 2019).

The 3D Cell Atlas add‐on for MorphoGraphX (Montenegro‐Johnson et al., 2015) was used to identify cell types within the radially symmetric root up to the pericycle (Stamm et al., 2017), and verified by manual inspection. Cell types were identified using the radial coordinate system provided by 3DCellAtlas (Montenegro‐Johnson et al., 2015; Stamm et al., 2017). This cell identity information was in turn used to annotate the identity of cell interfaces based on their adjacent cell type(s). Postprocessing in MorphoGraphX such as cell segmentation, mesh production, and cell type identification takes in total a number of hours depending on the sample; however, it could potentially be even faster as the pipeline is further refined.

### Quantification of plasmodesmata pit field signal on cell interfaces

Following 3D cell segmentation and mesh generation, signal from the plasmodesmata pit field z‐stack was integrated directly onto the 3D polygonal cell surface mesh using the Project Signal function in MorphoGraphX (Strauss et al., 2022; Yoshida et al., 2019). In this way, the spatial distribution of plasmodesmata pit field signal was directly embedded onto cell interface meshes, facilitating the multi‐scale quantification of pit field abundance, density, and patterning at cell interface‐specific resolution using MorphoGraphX.

The z‐stack containing the plasmodesmata pit field marker fluorescence was loaded into MorphoGraphX together with the mesh describing 3D cellular geometries and interfaces generated following segmentation. Signal from the plasmodesmata pit field stack was projected onto the mesh using the “Project Signal” function in MorphoGraphX, using a projection distance of 0.5 μm on either side of the mesh surface.

After plasmodesmata pit field signal was projected onto the mesh, background signal was removed using a low trim threshold. Compensation of photobleaching and signal decay in the inner cell layers was performed by decreasing the threshold trim into the inner cell layers based on cell annotations from 3DCellAtlas. After the trim, pit field signal was binarized by setting all remaining signal on the interface to the maximum voxel value. This defined where plasmodesmata pit field signal was either present or absent.

The mesh containing the pit field signal projected onto it was then used to calculate (1) Total Signal: the total amount of pit field signal on each interface, and (2) Signal Density: the density of pit field signal on each interface (Total Signal divided by interface surface area), and (3) the spatial distribution of signal using Moran's I. A separate mesh using a cube size of 0.2 was used for spatial analyses using Moran's I. Cell connectivity and interface size were calculated as previously described using the 3DCellAtlas plugin in MorphoGraphX (Jackson et al., 2017, 2019).

Following the analysis of plasmodesmata pit field signal on mesh interfaces in MorphoGraphX, a table containing extracted values was exported for statistical analyses. This subsequent export of the quantification of pit field signal is fairly rapid (taking a number of minutes, depending on the sample size and number of cells) and produces a CSV file which can be easily analyzed in programs such as excel. Cell interfaces smaller than 3 μm^2^ were removed. Pit field signal on cell interfaces was normalized by dividing these values by the sum of Total Signal on all interfaces of the sample to support equal signal intensity across samples, at the cost of comparing signal intensity between samples. This enabled the relative distribution of plasmodesmata pit fields between samples to be compared. Pit field signal was therefore reported as a proportion of the Total Signal across cell interfaces in the tissue such that values for all interfaces added up to a total of 1. This expressed as a percentage to represent the proportion of plasmodesmata pit field signal that was allocated to a particular cell interface type out of all the pit field signal recorded from the sample.

## AUTHOR CONTRIBUTIONS

GVD and GWB designed the research; GVD performed research; JJP, PL, MS, and RSS contributed new computational tools and genetic resources; GVD, RSS, EB and GWB analyzed data; GVD and GWB wrote the paper with input from coauthors.

## CONFLICTS OF INTEREST

The authors declare no competing interests.

## Supporting information


**Figure S1.** (A) Average total absolute signal intensity collected on different wild‐type root meristem cell interfaces in Arbitrary Intensity Units in each the meristem and transition zone of the root. (B) Same as (A) showing PD signal density in Arbitrary Intensity Units. Asterisks following *t*‐tests indicate significance levels of *0.01, **0.005, and ***0.001.
**Figure S2.** (A) Average total absolute signal intensity collected on different *bri1‐6* root meristem cell interfaces in Arbitrary Intensity Units. (B) Average signal density collected on different *bri1‐6* root meristem cell interfaces in Arbitrary Intensity Units.
**Figure S3.** Phenotyping of the UBQ10::SP‐mCherry‐PDCB1 line. (A) Number of days to flowering in the wild‐type and UBQ10::SP‐mCherry‐PDCB1 line. (B) Root length in the wild type and UBQ10::SP‐mCherry‐PDCB1 line. No significant differences were detected.

## Data Availability

Data are available on request from the authors.
