# Supplementary material for: Quantification of cell‐type‐specific plasmodesmata distribution in Arabidopsis roots reveals spatial and patterning dynamics
Source: Plant J. 2026 Feb 20;125(4):e70726. doi: 10.1111/tpj.70726 (PMC12922733; doi:10.1111/tpj.70726)
Supplement: Supplementary file 1 — Figure S1. (A) Average total absolute signal intensity collected on different wild‐type root meristem cell interfaces in Arbitrary Intensity Units in each the meristem and transition zone of the root. (B) Same as (A) showing PD signal density in Arbitrary Intensity Units. Asterisks following t‐tests indicate significance levels of *0.01, **0.005, and ***0.001. Figure S2. (A) Average total absolute signal intensity collected on different bri1‐6 root meristem cell interfaces in Arbitrary Intensity Units. (B) Average signal density collected on different bri1‐6 root meristem cell interfaces in Arbitrary Intensity Units. Figure S3. Phenotyping of the UBQ10::SP‐mCherry‐PDCB1 line. (A) Number of days to flowering in the wild‐type and UBQ10::SP‐mCherry‐PDCB1 line. (B) Root length in the wild type and UBQ10::SP‐mCherry‐PDCB1 line. No significant differences were detected. [file TPJ-125-0-s001.docx]

**Supplementary Data**

**Quantification of Cell-Type-Specific Plasmodesmata distribution in *Arabidopsis* roots reveals spatial and patterning dynamics**

**AUTHORS**

Gwendolyn V. Davis^1^, Jan J. Pavlou^1^, Patrick Li^2^, Marija Smokvarska^2^, Richard S. Smith^3^, Emmanuelle Bayer^2^, George W. Bassel^1,*^

**Supplementary Figure 1.** (A) Average total absolute signal intensity collected on different wild-type root meristem cell interfaces in Arbitrary Intensity Units in each the meristem and transition zone of the root. (B) Same as (A) showing PD signal density in Arbitrary Intensity Units. Asterisks following *t*-tests indicate significance levels of *0.01, **0.005, and ***0.001.

**
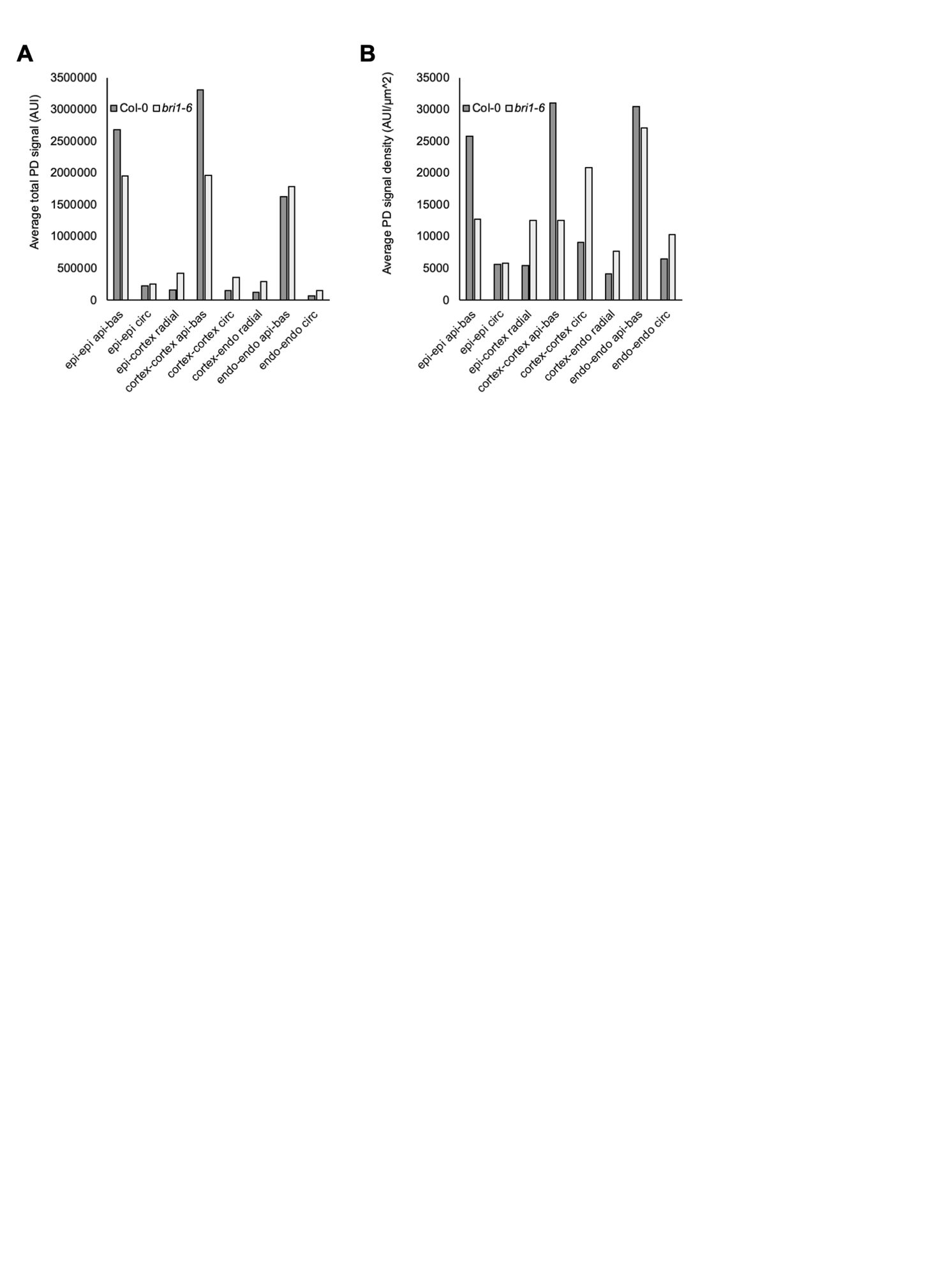
**

**Supplementary Figure 2** (A) Average total absolute signal intensity collected on different *bri1-6* root meristem cell interfaces in Arbitrary Intensity Units. (B) Average signal density collected on different *bri1-6* root meristem cell interfaces in Arbitrary Intensity Units.


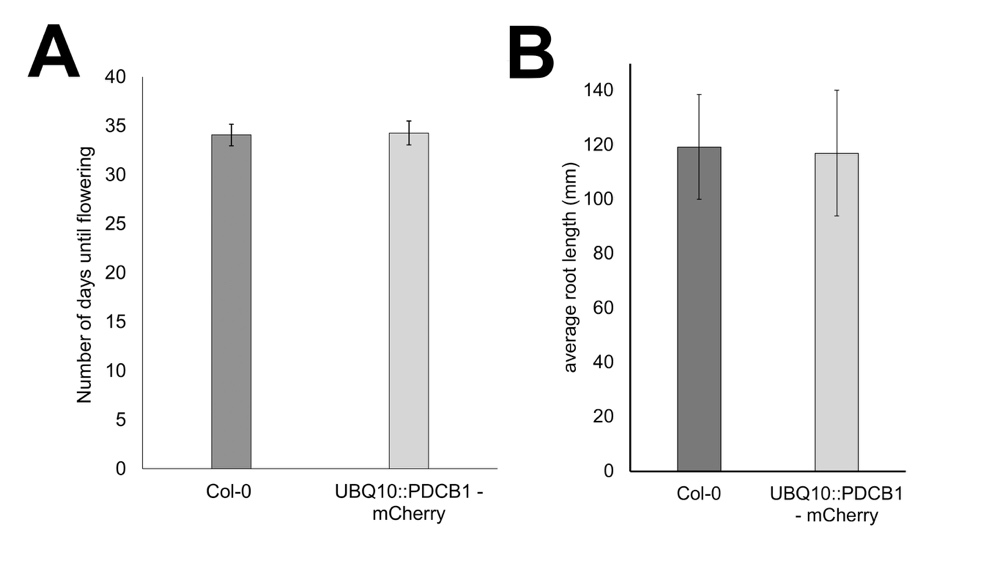


**Supplementary Figure 3.** Phenotyping of the UBQ10::SP-mCherry-PDCB1 line. (A) Number of days to flowering in the wild-type and UBQ10::SP-mCherry-PDCB1 line. (B) Root length in the wild type and UBQ10::SP-mCherry-PDCB1 line. No significant differences were detected.
